# Impact of a Dedicated Emergency Medicine Teaching Resident Rotation at a Large Urban Academic Center

**DOI:** 10.5811/westjem.2015.12.28977

**Published:** 2016-03-02

**Authors:** James Ahn, Andrew Golden, Alyssa Bryant, Christine Babcock

**Affiliations:** * University of Chicago, Department of Emergency Medicine, Chicago, Illinois; † Pritzker School of Medicine, Chicago, Illinois; ‡ Emory University Hospital, Department of Emergency Medicine, Atlanta, Georgia

## Abstract

**Introduction:**

In the face of declining bedside teaching and increasing emergency department (ED) crowding, balancing education and patient care is a challenge. Dedicated shifts by teaching residents (TRs) in the ED represent an educational intervention to mitigate these difficulties. We aimed to measure the perceived learning and departmental impact created by having TR.

**Methods:**

TRs were present in the ED from 12pm–10pm daily, and their primary roles were to provide the following: assist in teaching procedures, give brief “chalk talks,” instruct junior trainees on interesting cases, and answer clinical questions in an evidence-based manner. This observational study included a survey of fourth-year medical students (MSs), residents and faculty at an academic ED. Surveys measured the perceived effect of the TR on teaching, patient flow, ease of procedures, and clinical care.

**Results:**

Survey response rates for medical students, residents, and faculty are 56%, 77%, and 75%, respectively. MSs perceived improved procedure performance with TR presence and the majority agreed that the TR was a valuable educational experience. Residents perceived increased patient flow, procedure performance, and MS learning with TR presence. The majority agreed that the TR improved patient care. Faculty agreed that the TR increased resident and MS learning, as well as improved patient care and procedure performance.

**Conclusion:**

The presence of a TR increased MS and resident learning, improved patient care and procedure performance as perceived by MSs, residents and faculty. A dedicated TR program can provide a valuable resource in achieving a balance of clinical education and high quality healthcare.

## INTRODUCTION

In academic emergency departments (ED), formalizing the process to ensure high-quality clinical education for residents and medical students (MS) while also maintaining quality healthcare delivery is difficult. Achieving this balance has been increasingly challenging with ED crowding and a decline in bedside teaching practices. Compared to the 1960s when bedside teaching was common and comprised 75% of total teaching time, current estimates find that bedside teaching accounts for 17% of total teaching.^1^,^2^ While attending physicians believe bedside teaching to be effective, many cite time constraints as a frequent barrier.^3^ With the decline of bedside teaching, learner instruction in the ED has become challenging.

ED crowding compounds this challenge; studies have shown mixed results on the effect increasing patient volumes have on MS and resident teaching.^4^–^9^ To combat this issue, a number of institutions have implemented teaching attending physicians.^10^,^11^ Students and residents generally perceived a positive impact on bedside instruction as a results of these programs. Additionally, many institutions have developed residents-as-teacher (RAT) programs to improve clinical instruction by residents with variable success. While RAT programs have been developed in the ED, there are no outcomes reported on these interventions.^12^ A systematic review concluded RAT programs led to increased teaching skills and positive changes in RAT participants’ attitudes and perceptions about education.^13^ However, no study has examined the perceptions of non-participant ED personnel such as attending physicians, residents, and MSs of the presence of a dedicated teaching resident (TR) on learning and ED flow. Additionally, there is no literature that describes outcomes of a dedicated TR role that focuses only on teaching learners without the compounding variable of simultaneous direct clinical responsibilities.

The purpose of this study was to measure ED personnel’s perception on learning and departmental impacts by having a dedicated non-clinical TR in the ED at an urban, academic hospital. We hypothesized that with the presence of the TR, MSs and emergency medicine (EM) residents would indicate greater satisfaction with their learning experiences and ease of performing procedures and residents and attendings would perceive improved patient flow, patient care, and continuity of care with the presence of a dedicated non-clinical TR.

## METHODS

### Role of the Teaching Resident

The TR role is part of the curriculum for EM trainees at an academic three-year training program. The residency funded the development and staffing of the TR. Post-graduate year (PGY) 2/3 residents assumed the role of the TR; trainees successfully completed a RAT curriculum consisting of didactics and simulation prior to serving in this role. TRs were present in the ED during the busiest hours of operation (12pm to 10pm daily). PGY-2 residents worked two TR shifts per week during their community ED rotations (in addition to 16 standard clinical shifts), and PGY-3 residents worked five TR shifts as part of their “Education/Administration Rotation.” (No other shifts are required during this rotation.) During this time the TR’s only clinical responsibility was as the on-call flight physician for the hospital’s aeromedical transport program. The primary role was fulfilling the following teaching responsibilities: assisting and teaching procedures in the ED, preparing “chalk talks” for learners at the beginning of each shift, and instructing MSs and junior residents on interesting or difficult cases. The TR did not have an individual patient load, protecting their time to fulfill their teaching responsibilities. All 32 out of 32 residents eligible for the role participated.

### Study Design

This was an observational study from June 2012 to July 2013 involving the administration of a survey at an academic ED. Surveys were administered to fourth-year MSs performing their required EM clerkship (71 potential respondents), resident trainees in the EM program, including those serving as TR (48 potential respondents), and EM faculty (12 potential respondents). The institutional review board approved this study as consent exempt.

### Survey Content and Administration

We developed and piloted a survey instrument; content validity was established via an iterative process of review by EM education experts’ revision and piloting with 10 residents, five faculty members, and five MSs who were representative of the intended audience. Items less relevant for specific audiences were deleted, and wording changes were made as needed to reflect differences between MSs, residents, and faculty. Response process was established by reviewing feedback from the pilot implementation and by conducting a read-aloud session among the investigators. The survey included multiple-choice and free-text items. Each survey included either 13 or 14 statements requiring a response characterizing the study subjects’ perceptions of resident and MS teaching, patient flow, ease of procedures, and clinical care with and without the presence of the TR. Surveys were distributed using SurveyMonkey Inc. (Palo Alto, CA). These surveys were administered to each of the three subject groups, though the content of each group’s survey was slightly different. The link to the survey was distributed via email, allowing an anonymous response. Survey completion was optional with no consequences associated with completion. Only one response from each participant was requested. Two separate follow-up emails were sent to non-responders. The software tracked bounced emails and allowed invitees to opt out. Surveys for MSs, residents and faculty are attached in the Appendix A, B, and C, respectively.

### Data Analysis

In comparative statements (those requiring a response on a five-point scale ranging from “Poor” to “Excellent”), responses were converted to ordinal numbers (i.e. 1 = “Poor,” 5 = “Excellent”) for analysis. We calculated the mean and standard deviation. Additionally, we reported the difference between the means with and without the TR and the 95% percent confidence interval. Significance was determined in comparing statements with and without the presence of the TR using unpaired, two-tailed Student’s t-tests. We considered a p-value of less than 0.05 statistically significant. In statements requiring a response on a five-point Likert scale, the number of responses for “Somewhat Agree” and “Agree” were combined and reported as the percentage and absolute number of respondents indicating agreement. We performed all statistical analyses using Stata 12.1 (StataCorp, College Station, TX).

## RESULTS

The survey given to MSs yielded a 56% (40/71) response rate. MSs perceived a greater ease of procedures (Table 1, 2.23 vs. 4.33, p<0.001) and reported seeing fewer patients with the presence of the TR (Table 1, 4.12 vs 2.60, p<0.001). No statistical differences were found between their perception of their overall learning or the number of procedures they performed with the addition of the TR. Thirty students (75%) agreed or somewhat agreed they had a better overall educational experience during their clerkship with the TR, and 33 (82.5%) agreed or somewhat agreed the TR was a valuable educational experience. Eighteen students (45%) agreed or somewhat agreed the TR helped meet the needs of the fields into which they were matching (Table 2a).

The survey given to EM residents yielded a 77% (37/48) response rate. Residents perceived improved patient flow (2.24 vs 2.97, p<0.001), ease of procedures (2.76 vs 4.32, p<0.001), and MS learning (3.22 vs 4.25, p<0.001) with the presence of the TR (Table 3). Twenty-eight residents (75.6%) agreed or somewhat agreed the TR’s presence improved overall patient care (Table 2a/b). Nineteen residents (51.3%) agreed or somewhat agreed the TR improved the continuity of care. Thirty-one residents (83.8%) agreed or somewhat agreed the TR’s availability in the ED increased learning for the resident. Additionally, 28 residents (75.6%) agreed or somewhat agreed the TR added value to the ED team. Finally, isolating data gathered from residents who did not serve as the TR (PGY-1s) demonstrated that their perceptions were representative of the data gathered from all residents (Table 2a/b).

The survey given to EM attending physicians yielded a 75% (8/12) response rate. Attending physicians perceived significant increases in resident (3.38 vs 4.38, p<0.01) and MS learning with the TR (3.13 vs 4.50, p<0.01) (Table 4). They did not perceive change with patient flow through the department with the addition of the TR. All responding attending physicians (100%) agreed the TR aided with procedures in the department. Seven attending physicians (87.5%) agreed or somewhat agreed the TR improved patient care and 6 (75%) believed the TR added value to the ED team. Four (50%) believed the TR improved continuity of care in the ED (Table 2a).

We did not analyze qualitative results from the free-text portion of the survey secondary to the lack of a robust response rate and content.

## DISCUSSION

The addition of a dedicated TR without clinical duties at an urban, academic medical center ED improved perceptions of resident learning, procedural ease, and patient care by fourth-year MSs, EM residents, and EM attending physicians, validating some of our original hypotheses. A dedicated teaching attending program similarly improved perceptions of bedside teaching among residents and faculty.^10^ We believe a TR program such as this can help ballast a strong clinical education for learners in the ED by offering individual instruction and observation at academic institutions across the country, especially in institutions unable to offer dedicated teaching attending shifts.

As bedside teaching declines, dedicated TR shifts or rotations can help to improve the quality and quantity of bedside teaching for learners, especially resident trainees. MSs did not perceive the TR to enhance their overall learning, though they indicated the TR was overall a valuable educational experience. In a similar study, Hill et al. identified consistent findings after students instructed by a teaching-trained resident did not have superior outcomes to those who did not have a trained resident on an objective measure of clinical performance in their surgery clerkship.^14^

A majority of attending physicians and residents perceived the TR helped improve the quality of patient care within the ED, though this was unable to be objectively assessed. We hypothesize this perception is due to the direct impact of teaching procedures, critical thinking skills, and medical knowledge to learners. Many of our results focus on level one of Kirkpatrick’s model of evaluating training programs;^15^ assessing higher levels of training and evaluation would be of great interest in future studies of dedicated TR programs.

There were many perceived benefits from the addition of the TR in our institution. One unintended consequence of the TR for MSs during their EM clerkship was a perceived decrease in the number of patients seen. However, we are unclear if this is actually a negative consequence, as the goal of the MS rotation is to learn the basic tenets of EM. We speculate that the decreased number of patients is secondary to TRs spending more time instructing students on their current patients.

### Limitations

While this study shows the perceived effects of a TR on the learning and flow of an ED, it does have some limitations. At an institutional level, each RAT program has the ability to individually define the goals and objectives for the rotation within the ED; therefore, the perceived benefits we detected in our study may not be seen at different institutions. The sample size in this study, especially that of attending physicians, and the response rate of the MS group also limits the potential generalizability. Additionally, because we chose only to survey faculty, residents, and students, we considered the possibility for coverage error, as we did not seek other important stakeholders’ perceptions, such as ED nursing or staff. Also, the administration of the survey was not prior to the implementation of the TR; rather, it asked respondents to compare items before and after the implementation of this program, potentially introducing bias. Further, we were unable to determine if the perceptions detected in our results can actually be translated to objective educational and clinical outcomes in the ED. Also, the TR staffed the busiest hours of the ED and it is possible the position would be perceived as more or less impactful at other times of the day. Finally, this is a relatively small single institution study.

In order to continue demonstrating the clinical and educational impact of a dedicated TR, subsequent studies will have to measure Kirkpatrick level 2–4 outcomes among stakeholders in the ED. Additionally, future investigations can also focus on the potential benefit of a dedicated TR rotation to the resident serving in this role.

## CONCLUSION

This study is the first to examine the perceptions of a dedicated non-clinical TR program on the workings of an ED, in addition to the learning for MSs and residents in this environment. While more research is warranted to examine how these perceptions manifest in educational and clinical practice, this work demonstrates TRs are received with high acceptability among fourth-year MSs, EM residents, and EM attending physicians. Additionally, our study demonstrates a cost-benefit ratio for the addition of a TR, as the addition of a dedicated teaching position offers increased opportunities for education without impairing perceived patient flow in the ED. Instituting more programs such as this may help to encourage quality medical education for learners in the ED, or any challenging clinical environment, at busy academic medical centers.

## Supplementary Information







## Figures and Tables

**Table 1:** Medical student evaluation of emergency medicine clerkship experiences with and without the presence of the teaching resident (TR). Five-point scale responses were converted to ordinal numbers where 1=“Poor” and 5=“Excellent”. Values are reported as mean (SD). P values were determined by a two-tailed, unpaired Student’s t-test. The response rate was 40/71 (56%).

	Without TR	With TR	Difference [95% CI]	P-value
Overall learning	3.50 (0.94)	3.93 (1.11)	0.43 [−0.02, 0.88]	NS
Ease of procedures	2.23 (1.10)	4.33 (1.10)	2.10 [1.61, 2.59]	p<0.001
Number of procedures	3.02 (1.00)	2.76 (0.97)	−0.23 [−0.70, 0.16]	NS
Number of patients	4.12 (0.94)	2.60 (1.01)	−1.52 [−1.95, −1.10]	p<0.001

**Table 2a:** Subject agreement with characteristics of the teaching resident (TR) position and its effects on the emergency department (ED) - overall view. Values reported as percentage (absolute number) of responses in each response option.

	Disagree	Somewhat disagree	Neutral	Somewhat agree	Agree
Emergency medicine attending physicians					
Improves patient care	0% (0)	0% (0)	12.5% (1)	62.5% (5)	25% (2)
Improves continuity of care	12.5% (1)	12.5% (1)	25% (2)	25% (2)	25% (2)
Aids with procedures	0% (0)	0% (0)	0% (0)	0% (0)	100% (8)
Adds value to the ED team	0% (0)	0% (0)	25% (2)	37.5% (3)	37.5% (3)
Emergency medicine residents					
Improves patient care	0% (0)	5.4% (2)	18.9% (7)	29.7% (11)	45.9% (17)
Improves continuity of care	16.2% (6)	13.5% (5)	18.9% (7)	18.9% (7)	32.4% (12)
Improves learning for the resident	2.7% (1)	2.7% (1)	10.8% (4)	29.7% (11)	54.1% (20)
Adds value to the ED team	0% (0)	8.1% (3)	16.2% (6)	29.7% (11)	45.9% (17)
Emergency medicine residents (Non-TRs)					
Improves patient care	0% (0)	8.3% (1)	16.7% (2)	25% (3)	50% (6)
Improves continuity of care	8.3% (1)	16.7% (2)	25% (3)	41.7% (5)	8.3% (1)
Improves learning for the resident	0% (0)	0% (0)	8.3% (1)	25% (3)	66.7% (8)
Adds value to the ED team	0% (0)	8.3% (1)	16.7% (2)	25% (3)	50% (6)
Fourth year medical students					
Better experience with TR	2.5% (1)	7.5% (3)	15% (6)	32.5% (13)	42.5% (17)
Meets needs for field of interest	10% (4)	10% (4)	35% (14)	27.5% (11)	17.5% (7)
Valuable educational experience	2.5% (1)	5% (2)	10% (4)	32.5% (13)	50% (20)

**Table 2b:** Subject agreement with characteristics of the teaching-resident (TR) position and its effects on the emergency department (ED) – comparison response view. Values reported are percentage (absolute number) of respondents indicating “somewhat agree” or “agree” on a Likert scale. “#” indicates this group was not asked this question on their survey.

	EM attending physicians	EM residents	EM residents (Non-TRs)	Fourth year medical students
Improves patient care	87.5% (7)	75.7% (28)	75% (9)	#
Improves continuity of care	50% (4)	51.4% (19)	50% (6)	#
Aids with procedures	100% (8)	#	#	#
Adds value to the ED team	75% (6)	75.7% (28)	75% (9)	#
Improves learning for the resident	#	84.8% (31)	91.7% (11)	#
Better experience with TR	#	#	#	75% (30)
Meets needs for field of interest	#	#	#	45% (18)
Valuable educational experience	#	#	#	82.5% (33)

*EM,* emergency medicine

**Table 3:** Emergency medicine resident evaluation of emergency department experiences with and without the presence of the teaching resident (TR). Five-point scale responses were converted to ordinal numbers where 1=“Poor” and 5=“Excellent”. Values are reported as mean (SD). P-values were determined by a two-tailed, unpaired Student’s t test. The response rate was 37/48 (77%).

	Without TR	With TR	Difference [95% CI]	P-value
Patient flow	2.24 (0.72)	2.97 (0.96)	0.73 [0.34,1.12]	p<0.001
Ease of procedures	2.76 (0.54)	4.32 (0.81)	1.55 [1.24,1.87]	p<0.001
Medical student learning	3.22 (0.87)	4.25 (0.69)	1.03 [0.66,1.40]	p<0.001

**Table 4:** Emergency medicine attending physician evaluation of emergency department experiences with and without the presence of the teaching resident (TR). Five-point scale responses were converted to ordinal numbers where 1=“Poor” and 5=“Excellent”. Values are reported as mean (SD). P-values were determined by a two-tailed, unpaired Student’s t-test. The response rate was 8/12 (75%).

	Without TR	With TR	Difference [95% CI]	P-value
Patient flow	2.50 (0.76)	3.13 (0.99)	0.63 [−0.32, 1.57]	NS
Resident learning	3.38 (0.52)	4.38 (0.74)	1.00 [0.31, 1.69]	p<0.01
Medical student learning	3.13 (0.83)	4.50 (0.76)	1.38 [0.52, 2.23]	p<0.01
