# Supplementary material for: Impact of a Dedicated Emergency Medicine Teaching Resident Rotation at a Large Urban Academic Center
Source: West J Emerg Med. 2016 Mar 2;17(2):143–8. doi: 10.5811/westjem.2015.12.28977 (PMC4786233; doi:10.5811/westjem.2015.12.28977)
Supplement: Supplementary file 1 [file wjem-17-143-s001.pdf]

1. Default Section

1. Where did you do your ER rotation?

- ☐ University of Chicago
- ☐ Lutheran General
- ☐ Evanston Hospital

2. What field of medicine are you going into?

- ☐ Anesthesiology
- ☐ Dermatology
- ☐ Emergency Medicine
- ☐ Internal Medicine
- ☐ Family Medicine
- ☐ General Surgery
- ☐ Neurology
- ☐ OB/Gyn
- ☐ Ophthalmology
- ☐ Orthopedic Surgery
- ☐ ENT
- ☐ Pathology
- ☐ Pediatrics
- ☐ Psychiatry
- ☐ Radiation Oncology
- ☐ Radiology
- ☐ Urology

3. Rate your learning with the teaching resident

- ○ 1 - Poor
- ○ 2 - Below average
- ○ 3 - Average
- ○ 4 - Above average
- ○ 5 - Excellent

4. Rate your learning without the teaching resident

- ○ 1 - Poor
- ○ 2 - Below average
- ○ 3 - Average
- ○ 4 - Above average
- ○ 5 - Excellent

5. It is easier performing procedures with the teaching resident

- 1 - Disagree
- 2 - Somewhat disagree
- 3 - Neutral
- 4 - Somewhat agree
- 5 - Agree

6. It is easier performing procedures without the teaching resident

- 1 - Disagree
- 2 - Somewhat disagree
- 3 - Neutral
- 4 - Somewhat agree
- 5 - Agree

7. Rate the number of procedures performed with the teaching resident

- 1 - None
- 2 - Below Average
- 3 - Average
- 4 - Above average
- 5 - Many

8. Rate the number of procedures performed without the teaching resident

- 1 - None
- 2 - Below average
- 3 - Average
- 4 - Above average
- 5 - Many

9. Rate your number of patient's seen with the teaching resident

- 1 - None
- 2 - Below average
- 3 - Average
- 4 - Above average
- 5 - Many

10. Rate the number of patient's seen without the teaching resident

- 1 - None
- 2 - Below average
- 3 - Average
- 4 - Above average
- 5 - Many

11. You had a better experience in the ED with the teaching resident overall

- 1 - Disagree
- 2 - Somewhat disagree
- 3 - Neutral
- 4 - Somewhat agree
- 5 - Agree

12. The teaching resident meets the needs of your field of interest (ie, you are going into orthopedic surgery and you practiced joint reduction).

- 1 - Disagree
- 2 - Somewhat disagree
- 3 - Neutral
- 4 - Somewhat agree
- 5 - Agree

13. The teaching resident is a valuable educational experience

- 1 - Disagree
- 2 - Somewhat disagree
- 3 - Neutral
- 4 - Somewhat agree
- 5 - Agree

14. Comments or changes suggested for the teaching resident experience
