# Supplementary material for: Impact of a Dedicated Emergency Medicine Teaching Resident Rotation at a Large Urban Academic Center
Source: West J Emerg Med. 2016 Mar 2;17(2):143–8. doi: 10.5811/westjem.2015.12.28977 (PMC4786233; doi:10.5811/westjem.2015.12.28977)
Supplement: Supplementary file 2 [file wjem-17-143-s002.pdf]

1. Default Section

1. Level of training during 2009-2010 academic year

- PGY-1
- PGY-2
- PGY-3

2. Rate the patient flow in the ED with the teaching resident

- 1 - Poor
- 2 - Below average
- 3 - Average
- 4 - Above average
- 5 - Excellent

3. Rate the patient flow in the ED without the teaching resident

- 1 - Poor
- 2 - Below average
- 3 - Average
- 4 - Above average
- 5 - Excellent

4. Rate the ease of the procedures with the teaching resident

- 1 - Poor
- 2 - Below average
- 3 - Average
- 4 - Above average
- 5 - Excellent

5. Rate the ease of procedures without the teaching resident

- 1 - Poor
- 2 - Below average
- 3 - Average
- 4 - Above average
- 5 - Excellent

6. Rate medical student learning with the teaching resident

- 1 - Poor
- 2 - Below average
- 3 - Average
- 4 - Above average
- 5 - Excellent

7. Rate medical student learning without the teaching resident

- 1 - Poor
- 2 - Below average
- 3 - Average
- 4 - Above average
- 5 - Excellent

8. The presence of the teaching resident improves continuity of care (sign-off, hand-offs during flights, etc.)

- 1 - Disagree
- 2 - Somewhat disagree
- 3 - Average
- 4 - Somewhat agree
- 5 - Agree

9. The presence of the teaching resident improves patient care

- 1 - Disagree
- 2 - Somewhat disagree
- 3 - Neutral
- 4 - Somewhat agree
- 5 - Agree

10. The presence of the teaching resident improves learning for the resident

- 1 - Disagree
- 2 - Somewhat disagree
- 3 - Neutral
- 4 - Somewhat agree
- 5 - Agree

11. Rate the overall value of the teaching resident to the MER team

- 1 - Poor
- 2 - Below average
- 3 - Average
- 4 - Above average
- 5 - Excellent

12. Participating as the Teaching Resident is a valuable educational experience

- 1 - Disagree
- 2 - Somewhat disagree
- 3 - Neutral
- 4 - Somewhat agree
- 5 - Agree

13. Comments or changes suggested for the teaching resident experience
