# Supplementary material for: Impact of a Dedicated Emergency Medicine Teaching Resident Rotation at a Large Urban Academic Center
Source: West J Emerg Med. 2016 Mar 2;17(2):143–8. doi: 10.5811/westjem.2015.12.28977 (PMC4786233; doi:10.5811/westjem.2015.12.28977)
Supplement: Supplementary file 3 [file wjem-17-143-s003.pdf]

1. Default Section

1. Rate the patient flow in the ED with the teaching resident

- 1 - Poor
- 2 - Below average
- 3 - Average
- 4 - Above average
- 5 - Excellent

2. Rate the patient flow in the ED without the teaching resident

- Poor
- Below average
- Average
- Above average
- Excellent

3. Rate resident learning with the teaching resident present

- Poor
- Below average
- Average
- Above average
- Excellent

4. Rate resident learning with the teaching resident absent

- Poor
- Below average
- Average
- Above average
- Excellent

5. Rate the medical student learning with the teaching resident

- Poor
- Below average
- Average
- Above Average
- Excellent

6. Rate the medical student learning without the teaching resident

- ☐ Poor
- ☐ Below average
- ☐ Average
- ☐ Above Average
- ☐ Excellent

7. The presence of the teaching resident improves patient care

- ☐ Disagree
- ☐ Somewhat disagree
- ☐ Neutral
- ☐ Somewhat agree
- ☐ Agree

8. The presence of the teaching resident does not improve patient care

- ☐ Disagree
- ☐ Somewhat disagree
- ☐ Neutral
- ☐ Somewhat agree
- ☐ Agree

9. The presence of the teaching resident improves continuity of care (sign-out, hand off's during flights, etc.)

- ☐ Disagree
- ☐ Somewhat disagree
- ☐ Neutral
- ☐ Somewhat agree
- ☐ Agree

10. The presence of the teaching resident does not improve continuity of care

- ☐ Disagree
- ☐ Somewhat disagree
- ☐ Neutral
- ☐ Somewhat agree
- ☐ Agree

11. The presence of the teaching resident aids with procedures

- ☐ Disagree
- ☐ Somewhat disagree
- ☐ Neutral
- ☐ Somewhat agree
- ☐ Agree

12. The presence of the teaching resident does not aid with procedures

- ☐ Disagree
- ☐ Somewhat disagree
- ☐ Neutral
- ☐ Somewhat agree
- ☐ Agree

13. Rate the overall value of the teaching resident to the MER team

- ☐ Poor
- ☐ Below average
- ☐ Average
- ☐ Above average
- ☐ Excellent

14. Comments or changes suggested for the teaching resident role
